# Violating statistical structure impairs detection of deviant and incidental events

**DOI:** 10.1016/j.isci.2026.116383

**Published:** 2026-06-17

**Authors:** Emma K. Ward, Nick Simpson, Clare Press

**Affiliations:** 1 Department of Experimental Psychology, UCL, 26 Bedford Way, London WC1H 0AP, UK; 2 Department of Imaging Neuroscience, UCL, 12 Queen Square, London WC1N 3AR, UK; 3 School of Psychological Sciences, Birkbeck, University of London, Malet Street, London WC1E 7HX, UK

**Keywords:** neuroscience, sensory neuroscience, psychology

## Abstract

Learning about the statistical structure of our environment is thought to shape perception, but it is unclear how. A recent theory suggests that percepts are initially biased toward the expected, with particularly unexpected observations triggering reactive sensory gain increases—balancing requirements for fast and accurate perception alongside reliable sensory estimates for model updating. Six experiments tested this account, where participants detected visual stimuli on the circumference of, and at the center of, a circle. Circumference stimuli followed a spatial or orientation regularity, which then changed abruptly. Bayesian changepoint modeling showed that hit rates were lower for all events following such disruption of the learned probabilistic structure (hereafter “surprise”). Performance recovery after one change took several trials but became immediate when changes were more frequent. These findings suggest broad perceptual facilitation of the expected regardless of latency, and we thus consider how models may be accurately updated when the world changes, despite poorer perception.

## Highlights

• After a surprising event, detection of all events is impaired for several seconds
• Impairments were found for features of the surprising event and concurrent events
• Contrary to expectations, impairments were exhibited at all latencies
• We discuss how models may be updated when the world changes despite poor perception

## Graphical abstract

**Figure:**
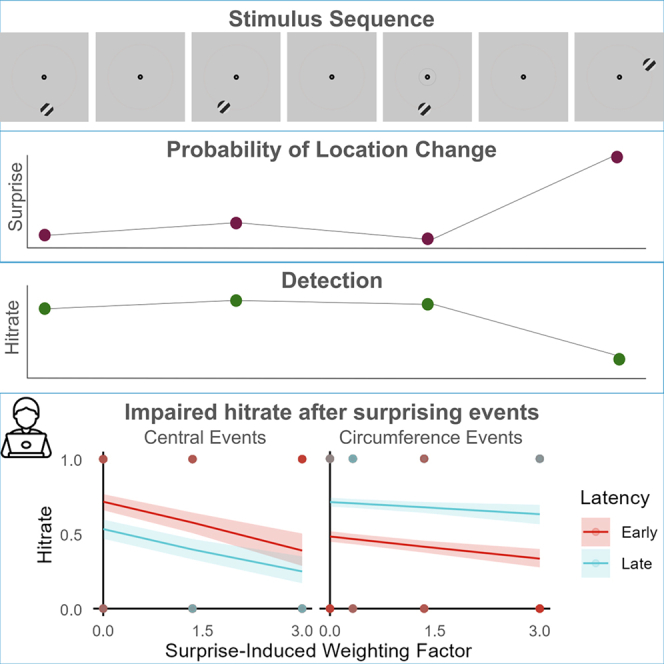

## Introduction

Bayesian theories of perception propose that our experiences reflect a precision-weighted combination of expectations and sensory input.^1^^,^^2^^,^^3^ When the regularities of our environment are stable, such prediction is adaptive in enabling efficient and accurate inference.^4^ For example, we may learn that the kettle is typically in the area next to the toaster in our kitchen, and we would usually be correct if perceiving it there in the face of noisy, rapidly acquired sensory input—such as when entering the kitchen in the dark, or when simultaneously holding a conversation.

When the regularities of the environment change, however, new observations should exert a greater influence on inference.^5^^,^^6^ Because surprising events often signal a change in the regularities of the environment, we likely need to form precise percepts in those surprising moments. Precise percepts will optimize accurate model updating. For instance, if the kettle has moved to another corner of the kitchen, we need to perceive this shift when we want to make tea, along with surrounding events like the presence of our housemate, which may provide an explanation for its shift. What mechanisms enable us to form such precise percepts and subsequently learn about the new state of the world?

One proposal is seen in cancellation perceptual accounts, which suggest that expected features are attenuated within input processing to render a percept that highlights surprising features.^7^^,^^8^ However, such a mechanism would act in opposition to that outlined in Bayesian accounts, fails to account for the additional and competing adaptive requirement for our perceptual system to optimize veridical experience given sensory noise,^4^^,^^9^ and is inconsistent with findings demonstrating poor perception of unexpected events relative to expected events.^4^^,^^10^^,^^11^ An account of how prediction shapes perception must be able to account for the multitude of neural and behavioral findings demonstrating both enhanced and reduced perceptual processing of the expected.^12^^,^^13^^,^^14^^,^^15^^,^^16^^,^^17^^,^^18^

A proposal for overcoming this problem of rendering percepts both veridical and informative requires the operation of two partially dissociable processes.^4^ Under this “opposing processes” proposal, we are initially biased to perceive what we expect via increasing the gain of expected channels. Such a mechanism generates best guesses rapidly that accurately reflect our world most of the time. However, if an event is out-of-line with learned estimates of the variability of the environment, a range of mechanisms may reactively improve perception of this surprising event to enable accurate model updating. One possibility is that phasic noradrenaline release increases sensory gain, enabling generation of precise percepts when the world changes. It is known that noradrenaline plays a crucial role in the increased learning rates observed when regularities in the environment are disrupted^19^^,^^20^ and that tonic higher noradrenaline increases the signal-to-noise ratio in the sensory thalamus.^21^ Under this account, sensory sensitivity is predicted to be higher following a surprising event after a short delay.

To test this account, we asked whether at early time points (<200 ms after the presentation of the stimulus), the detection of expected events is enhanced compared to unexpected events, and at later time points (≥200 ms), we see the opposite effect. We were particularly interested in determining whether detection changes following the unexpected would extend to all event types—specifically, whether detection improvements could be seen for both features of the surprising events themselves and other events presented in parallel, given the hypothesized mechanism that may increase sensory gain across channels. Importantly, based upon the theoretical account’s emphasis on surprising events exceeding a threshold of unexpectedness, we ensured that the surprising change in the regularities was extreme. To this end, our studies present a paradigm where the surprising events truly were out of line with learned distributional knowledge. This design is in contrast with the vast majority of studies into predictive perception, which present frequent changes or inform participants of changes in advance.

**Figure 1:**
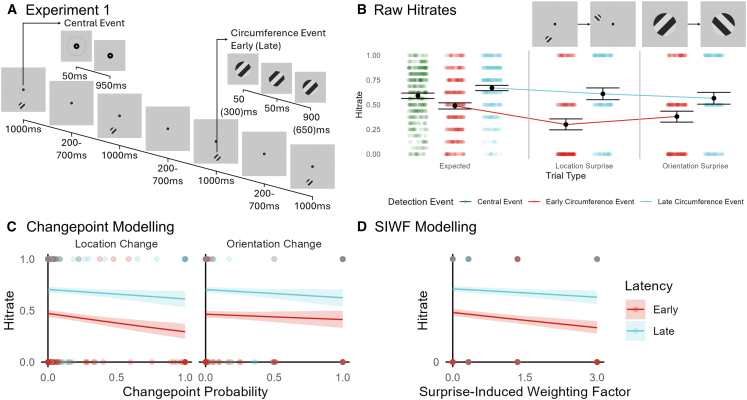
Experiment 1 methods and results (A) On 25% of trials, a gray ring appears around fixation (central event). On a separate 25% of trials, the circumference stimulus phase shifted for 50 ms at an early or late latency. (B) Raw hit rates of expected trials (pre-surprise) and surprise trials (first two after surprise). Location surprises occurred when the circumference stimulus changed location around the circumference by 90°. Orientation surprises occurred when the grating orientation switched from +45° to −45° (or vice versa). The hit rates are separated for central and for early and late circumference events. Black points refer to the mean hitrate with error bars representing the bootstrapped 95% confidence interval. (C) Marginal means from changepoint modeling of location change and orientation change, with changepoint probability on the *x* axis and circumference hit rate on the *y* axis. Shaded regions represent the 95% confidence intervals of the fixed-effects model predictions. (D) SIWF model marginal means, with modeled SIWF value on the *x* axis and circumference hit rate on the *y* axis. Phase shifts at 50 ms in red and 300 ms in blue. See also Figure S1.

Participants were presented with a display consisting of a red circle presented centrally on the screen (see Figure 1A). In experiment 1, small square grating stimuli were then presented at a variety of locations drawn from a truncated normal distribution on the circumference of the red circle, generating a small amount of expected uncertainty in stimulus location (for a similar paradigm used in learning studies, see O’Reilly et al.^22^ and Vaghi et al.^23^). Locations were drawn from the same truncated normal distribution for a first run of experimental trials, and orientation was also held constant. After this run, either the location distribution or the orientation of the gratings changed abruptly and without warning. Participants were instructed to respond if they detected a phase shift of these gratings presented on the circumference, which was presented on ∼25% of trials and occurred either early (50 ms) or later (300 ms) after grating onset. They were also instructed to respond if they detected the appearance of a stimulus at the center of the display (a small gray ring), which was presented on a further ∼25% of trials. This central task both encouraged participants to fixate the center of the display and, in subsequent studies, allowed us to probe how the expectedness of events at the circumference influenced the detection of “incidental” events at the center—i.e., events that were not those generating the surprise.

Experiment 1 was optimized to examine the detection of features of the surprising events themselves. Experiments 2 and 3 asked whether effects would be seen for other, centrally presented sensory events and were therefore optimized to examine the detection of these events. Experiment 4 studied whether these effects were feature specific. Experiment 5 investigated the detection of incidental events over the time course of a longer experiment with periodic changes, to determine whether the perceptual effects change as observers can learn to anticipate that the environmental regularities fluctuate. Finally, experiment 6 dissociated the effects of location repetition from location expectation. To pre-empt our findings, we find that—regardless of latency—surprise elicited by change on the circumference events resulted in impairments in detection of features of those circumference events, as well as events at the center. We therefore discuss how one may update our models of the world accurately when it changes in spite of uniformly poorer perception.

## Results

### Experiment 1

Experiment 1 asked whether, when events are presented that are inconsistent with learned variability within the environment, perception of features of those surprising events is enhanced at short delay (300 ms) relative to perception before any purported reactive processes could have improved perception (50 ms), and relative to when expected events are presented. Full methods are found in STAR Methods experiment 1.

Participants were presented with grating stimuli on the circumference of a red circle (Figure 1). They were asked to detect any central events (circle appearing around the fixation point; approx. 25% of trials) along with phase shifts on the circumference events (approx. 25% of trials). They pressed the F key in response to fixation events and the J key in response to circumference events.

During training and the first run of the main task, the locations of the circumference stimuli were drawn from a distribution with a stable mean and the gratings were presented at the same angle. At the start of the second run, either the location of the circumference stimuli would abruptly change by 90° (location change) or the orientation of the gratings would change by 90° (orientation change). On the first trial of the third run, the participants would be presented with the other category of change. The phase shifts could occur 50 or 300 ms after the onset of the circumference stimulus.

**Figure 2:**
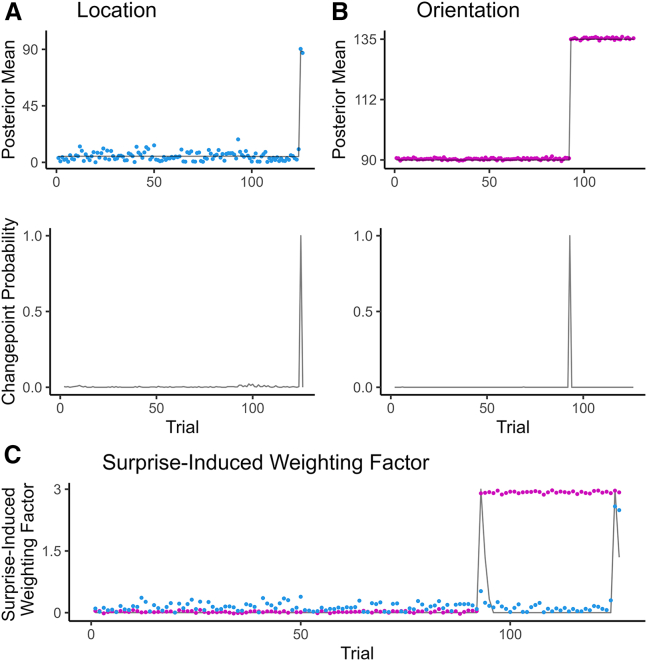
Modeled surprise predictors (A) Changepoint analysis for stimulus location for one example participant, with trials on the *x* axis. Practice trials are included in this demonstration where it is intended that the practice contributes to their beliefs concerning the statistics of the environment. This participant performed 60 practice trials (20 for central detection, 20 for circumference detection, and 20 more circumference detection as performance was poor) in addition to the 2 runs of 32 trials and the 2 trials in run 3. The *y* axes reflect estimated mean location (top) and estimated probability of a change in the distribution underlying the observed locations (bottom). (B) Changepoint analysis for stimulus orientation for one example participant, with trials on the *x* axis. The *y* axes reflect estimated mean orientation (top) and estimated probability of a change in the distribution underlying the observed orientation (bottom). (C) Modeled SIWF, which peaks on trials with changepoint probability above a certain threshold and decays gradually over the following 3 trials.

We calculated the probability of a change in the underlying statistics of the environment at each time point using a Bayesian changepoint analysis,^24^^,^^25^^,^^26^ for both the location (see Figure 2A) and the orientation (see Figure 2B) of the circumference stimuli. This changepoint probability was then entered into binomial generalized linear multi-level models as a predictor, to determine whether the probability that each trial represented a change in the underlying generative distribution, given the previously observed samples, predicted detection. We contrasted this analysis against one where the changepoint probabilities computed in the previous step triggered a process which peaked on the surprising trial and decayed over the subsequent trials, reflecting the possibility that any reactive changes in sensory gain may operate over a longer timescale (surprise-induced weighting factor [SIWF]).

Hit rate was computed for the circumference phase shifts and the central events separately, for each participant. Participants performed similarly on the two tasks and in a range which rendered floor or ceiling effects unlikely (see Table 1). We included also a comparison group of participants who did not receive detection events on critical trials and note that no effect of surprise was found on the false alarm rate (Figure S1). Additionally, reaction times were not found to be different between expected or surprising trials (see Figure S5 for reaction time analysis for all experiments, noting that no differences were found except for in experiment 4).

**Table 1:** Hit rates and false alarms across all six experiments

	Hit rate circumference event Phase shift	Hit rate circumference event Gray stimulus	Hit rate central event	False alarm rates
Experiment 1	55.3% (23.9%)	–	58.0% (27.4%)	0.96% (2.69%)
Experiment 2	–	84.4% (21.4%)	42.6% (31.8%)	5.63% (7.89%)
Experiment 3	–	87.5% (14.4%)	56.0% (28.4%)	4.81% (4.42%)
Experiment 4	–	80.4% (18.6%)	47.1% (31.1%)	5.71% (5.09%)
Experiment 5	–	89.5% (6.97%)	55.4% (26.2%)	1.89% (1.24%)
Experiment 6a	65.5% (21.6%)	–	66.2% (26.3%)	1.70% (1.99%)
Experiment 6b	–	89.4% (17.5%)	60.0% (34.1%)	4.45% (11.3%)

#### Changepoint probability

The primary predictors of interest were probability of a changepoint in location and in orientation, phase shift latency, and the interaction of latency with the changepoint probabilities (see Table S2 for full model output). Changepoint probability for stimulus location negatively predicted hit rate (log-odds = −0.56, confidence interval [CI] = −0.83 to −0.30, *z* = −4.13, *p* < 0.001), as did the latency of the phase shift (log-odds = −0.51, CI = −0.57 to −0.45, *z* = −16.64, *p* < 0.001), and the two terms did not interact (see Figure 1B for raw hit rates; Figure 1C; Table S2 for model parameters). Changepoint probability for stimulus orientation also negatively predicted hit rate (log-odds = −0.30, CI = −0.55–0.04, *z* = −2.29, *p* < 0.05), and this factor did not interact with phase shift latency (see Figure 1C; Table S2).

These findings demonstrate that surprise reduced detection of features of surprising events, both when that surprise was generated via a change in spatial location and a change in stimulus orientation. This reduced detection was exhibited both when the event to be detected was presented at 50 and 300 ms after stimulus onset.

#### SIWF

The primary predictors of interest were modeled SIWF, triggered by any changepoint probability of location or orientation that exceeded the threshold; phase shift latency; and the interaction of latency with the SIWF (see Table S3 for full model output).

Modeled SIWF also negatively predicted hit rate (log-odds = −0.16, CI = −0.22 to −0.09, *z* = −4.46, *p* < 0.001), as did the latency of the phase shift (log-odds = −0.50, CI = −0.56–0.45, *z* = 15.87, *p* < 0.001). Similarly to the changepoint probability analysis, the two terms did not interact (see Figure 1D; Table S3). The SIWF model (Akaike information criterion [AIC] = 8,320.9) outperformed the changepoint probability model (AIC = 8,324.8), indicating that our data are better explained by our modeled SIWF value than by the standard changepoint probability.

### Experiment 2

Experiment 1 showed reduced detection of features of surprising events themselves, where the surprise was elicited either via a change in spatial location or stimulus orientation. Such reduced detection was observed when the feature was presented at 50 and 300 ms after the surprising event onset, and the reduction persisted in the trials that followed. This finding was counter to the prediction that detection is improved after a short delay following surprise.

Experiment 2 therefore interrogated another possible way that perception may be optimized for model updating. Namely, that a change in the environment causes the observer to explore the environment for other cues that may act as predictor variables in a revised model. Such a process could result in poorer detection of features of the surprising stimulus itself but improved detection of other available cues. In order to be adaptive for inference, such redirection of resources is hypothesized to operate shortly after surprising event onset.^4^

To this end, experiment 2 tested whether detection of the central event is improved shortly after surprising events at the circumference, by modulating the time of these central events and determining the influence on perception. We made two other changes relative to experiment 1. First, the circumference stimuli were now filled black circles rather than square gratings because we did not need to present events with different onset latencies embedded in the surprising circumference stimuli. As a result, the circumference detection event was presentation of a gray rather than black circle. Second, the early events were now at 0 ms rather than 50 ms, and the late events at 200 ms rather than 300 ms. The early events could be presented at 0 ms as they did not require a comparison phase earlier in stimulus presentation like in experiment 1, and the later time window was chosen to coincide with a behavioral enhancement effect found in an earlier study.^13^ For full experimental details, see STAR Methods experiment 2.

If the surprising event generates improved detection of other events in the environment, we hypothesized an interaction between expectedness and latency, such that hit rate to the central event 200 ms after the onset of the surprising event would be higher than hit rate on expected trials and higher than hit rate at 0 ms after the onset of the surprise. The changepoint probability and SIWF were again used to interrogate these effects.

**Figure 3:**
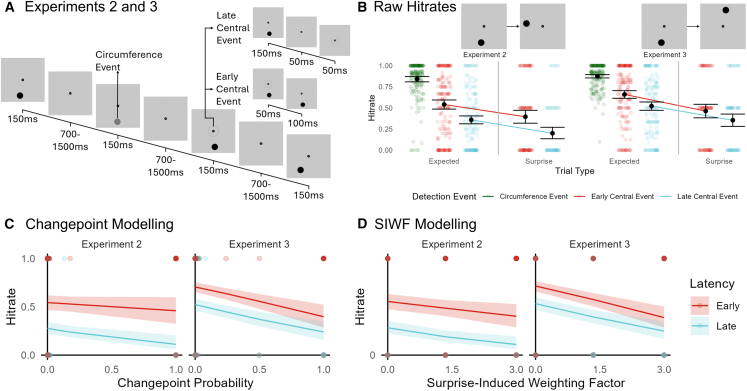
Experiment 2 and 3 methods and results (A) In experiments 2 and 3, the circumference stimuli were presented as solid black circles. On 25% of trials, the circumference stimulus was instead gray (circumference event). On a separate 25% of trials, the central fixation ring was presented for 50 ms, either concurrent with the circumference stimulus or 200 ms after. (B) Raw hit rates of expected trials (pre-surprise) and surprise trials (first two after surprise) for circumference, early central, and late central events. In experiment 2 the surprise was defined by a 90° change in circumference stimulus location, and in experiment 3, the location changed by 180°. Black points refer to the mean hitrate with error bars representing the bootstrapped 95% confidence interval. (C) Changepoint modeling of central events at 0 ms in red and 200 ms in blue. Shaded regions represent the 95% confidence intervals of the fixed-effects model predictions. (D) SIWF modeling of central events at 0 ms in red and 200 ms in blue.

Changepoint probability for circumference stimulus location negatively predicted hit rate to the central event (log-odds = −0.81, CI = −1.37 to −0.26, *z* = −2.86, *p* < 0.01); the latency of the central event positively predicted hit rate (log-odds = 0.57, CI = 0.45–0.69, *z* = 9.00, *p* < 0.001), and the two did not interact (see Figure 3C; Table S2). The surprise-induced weighting analysis gave similar results, negatively predicting hit rate (log-odds = −0.41, CI = −0.66 to −0.15, *z* = −3.14, *p* < 0.01), with latency of the central event a positive predictor (log-odds = 0.57, CI = 0.44–0.71, *z* = 8.53, *p* < 0.001), and no interaction (see Figure 3D; Table S3). Consistent with experiment 1, the SIWF-based model (AIC = 2,524.6) outperformed the changepoint probability model (AIC = 2,540.5).

The early events were thus easier to detect than the later events, in contrast with experiment 1 where the late events were detected better. These main effects are hard to interpret because they represent a number of differences. They most likely reflect that it is hard to detect phase shifts when the grating has only just been presented, whereas fixation events benefit from being presented simultaneously with the circumference events. More importantly, pertaining to the influence of expectation on detection, in line with experiment 1, Experiment 2 again found that surprise impaired detection—this time of the incidental events.

### Experiment 3

Experiment 2 demonstrated that surprise generated by a change in location of a circumference stimulus reduced hit rate to central events. This finding was counter to the idea that surprise may increase exploration or foraging, and hence improve detection of events elsewhere in the environment. Given that one possible explanation for this finding is that the shift in location in experiment 2 was not large enough to generate sufficient surprise, experiment 3 examined whether detection of events following surprise would indeed increase if the change in the environment was larger in magnitude.

Experiment 3 therefore replicated experiment 2 with a larger displacement of the circumference stimulus (180° rather than 90°). If we replicated experiment 2, we should see that surprise negatively predicts hit rate. However, if larger levels of surprise generate perceptual enhancement of events following surprise, we may see an increase in hit rate at 200 ms after the surprising event.

Findings were identical to experiment 2. Changepoint probability for stimulus location was a negative predictor (log-odds = −1.30, CI = −1.73 to −0.87, *z* = −5.90, *p* < 0.001), latency a positive predictor (log-odds = 0.39, CI = 0.27–0.50, *z* = 6.80, *p* < 0.001), and there was no interaction (see Figure 3C; Table S2). The SIWF negatively predicted hit rate (log-odds = −0.50, CI = −0.64 to −0.35, *z* = −6.79, *p* < 0.001), latency of the central event positively predicted hit rate (log-odds = 0.39, CI = 0.27–0.50, *z* = 6.50, *p* < 0.001), and the two terms did not interact (see Figure 3D; Table S3). Consistent with experiments 1 and 2, the SIWF-based model (AIC = 2,817.7) outperformed the changepoint probability model (AIC = 2,831.1).

### Experiment 4

Experiment 1 found that a surprising event at the circumference reduced detection of features of those surprising events, and experiments 2 and 3 found that it also reduced detection of other events in the environment—in this case, the central event. These findings demonstrate that surprise does not automatically increase the detection of features of surprising events, nor did it increase detection of other events in the environment—quite the opposite. Given that the circumference and central events were visually dissimilar, and performance may have been tuned to specific visual features, experiment 4 examined how a change in the location of the circumference event altered detection of an additional, visually similar, circumference event.

To this end, participants were presented with two circumference stimuli separated by 20°–40°. The surprising trial saw one of the stimuli move by 90° and the other remaining in the same location. For half of the participants, the detection event was presented on the circumference stimulus that remained at the expected location, and for the other half, it was presented on the stimulus at the new, unexpected location.

**Figure 4:**
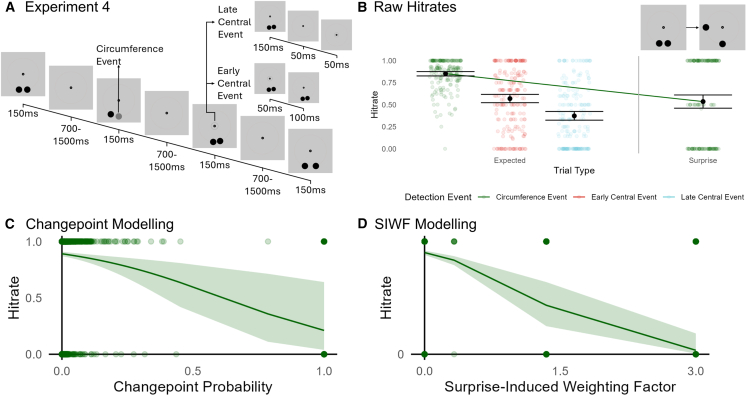
Experiment 4 methods and results (A) In experiment 4, two black circumference stimuli were presented concurrently. On 25% of trials, one of the circles would be gray (circumference event). On a separate 25% of trials, the central fixation ring would appear for 50 ms, either concurrently with the circumference stimuli or 200 ms after. (B) Raw hit rates of expected trials (pre-surprise) and surprise trials (first two after surprise) for circumference, early central, and late central events. The surprise trials were defined by one of the circumference stimuli moving by 90°. Black points refer to the mean hitrate with error bars representing the bootstrapped 95% confidence interval. (C) Changepoint modeling of circumference event hit rate. Shaded regions represent the 95% confidence intervals of the fixed-effects model predictions. (D) SIWF modeling of circumference event hit rate. See also Figure S2.

Changepoint probability for stimulus location again negatively predicted hit rate (log-odds = −3.342, CI = −5.31 to −1.53, *z* = 3.55, *p* < 0.001; see Figure 4C; Table S2), as did the SIWF (log-odds = −0.50, CI = −0.64 to −0.35, *z* = −6.079, *p* < 0.001; see Figure 4D; Table S3), consistent with experiments 1–3. Again consistent with experiments 1–3, the SIWF model (AIC = 1,993.3) outperformed the changepoint probability model (AIC = 2,109.2; unlike the other experiments reported here, participants were also slower to respond to the surprising events; see Figure S5). This experiment adds to the findings of experiments 2 and 3 by demonstrating that this effect is unaffected by the nature of the additional event: even when the same stimulus type is shown at both an expected and surprising location, detection of features of the surprising stimulus is impaired.

### Experiment 5

In experiment 5, we tested whether hit rate to hard-to-detect features of a central event increases shortly after the circumference stimulus abruptly changes in location but with more frequent changes. This paradigm was almost identical to experiments 2 and 3, with locations in each run drawn from a distribution either 90° or 180° from the previous run’s location and a new run every 10–20 trials. However, we now presented 32 runs rather than three. Therefore, we could determine how the aforementioned processes differ when participants can learn to anticipate that the location may change.

We also generated higher variability in the onset of the central event, such that it was presented from 0 to 300 ms relative to the onset of the circumference stimulus in increments of 50 ms. The greater range of latencies allowed us to determine whether there were any short-lived effects missed with the designs of experiments 1–4.

**Figure 5:**
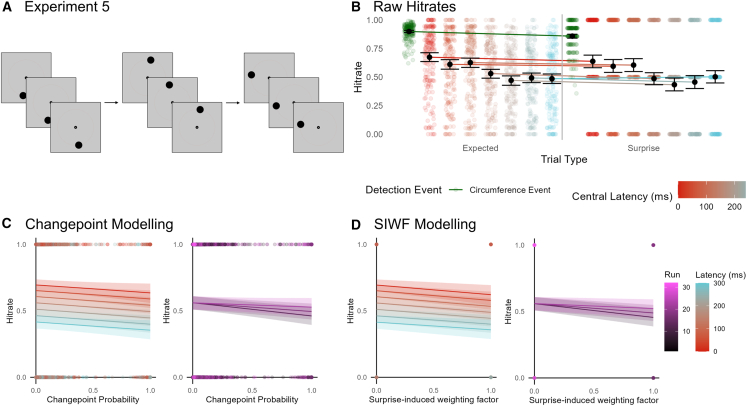
Experiment 5 methods and results (A) In experiment 5, the trials were identical to those in experiment 2. However, the mean location of the circumference stimuli changed every 10–20 trials for 32 runs. (B) Raw hit rates of expected trials (pre-surprise) and surprise trials (first two after surprise) for circumference events and central events with varying latencies. The surprise was defined as each time the mean location of the circumference stimulus changed. Black points refer to the mean hitrate with error bars representing the bootstrapped 95% confidence interval.[[parms resize(1),pos(50,50),size(200,200),bgcol(156)]]|PDF FILE NOT READY latency (left) or run number (right). Shaded regions represent the 95% confidence intervals of the fixed-effects model predictions. (D) Same as (C) but modeled with the SIWF.

Changepoint probability for circumference stimulus location significantly negatively predicted hit rate to the central event (log-odds = −0.28, CI = −0.54 to −0.02, *z* = −2.13, *p* < 0.05), and the latency of the central event negatively predicted hit rate to the central event (log-odds = −0.19, CI = −0.21 to −0.18, *z* = −24.43, *p* < 0.001; see Figure 5C; Table S3). The model including the interaction with run (AIC = 27,982.9) slightly outperformed the equivalent model without this interaction (AIC = 27,984.2), but the decreasing influence of changepoint probability on hit rate as the experiment progressed was only marginally significant (log-odds = 0.01, CI = −0.00–0.03, *z* = 1.83, *p* = 0.067).

Unlike experiments 1–4, interestingly, the optimal SIWF in this experiment was one which took a positive value only for the first trial in a new location and zero for all other trials (see Table S1). The SIWF negatively predicted hit rate to the central event (log-odds = −0.33, CI = −0.59 to −0.08, *z* = −2.56, *p* < 0.05), as did the latency of the central event (log-odds = −0.19, CI = −0.21 to −0.18, *z* = −25.16, *p* < 0.001), and the two did not interact (see Figure 5D; Table S4). The model including the interaction with run (AIC = 27,980.8) slightly outperformed the equivalent model without this interaction (AIC = 27,982.1), suggesting a decreasing influence of SIWF on hit rate over the time course of the experiment, but again the effect was marginal (log-odds = 0.01, CI = −0.00–0.03, *z* = 1.82, *p* = 0.069). The SIWF-based model (AIC = 27,980.8) performed similarly to the changepoint probability model (AIC = 27,982.9) in this study, unlike the others, in line with the fact that the best-fitting SIWF was one with immediate decay and thus more similar to the changepoint probability (note that they also differ in whether small deviations are included, but previous studies also found this feature unlikely to drive differences; see Table S1).

To summarize, experiment 5 tested whether hit rate to hard-to-detect central events accompanying surprise recovered differently when one could anticipate that circumference events *could* change. Like in experiments 1–4, hit rate reduced upon surprise, but unlike in these experiments, interestingly, hit rate recovered after one trial.

### Experiment 6

The current results show that the hit rate to both central and circumference stimuli deteriorates after a surprising location (or orientation) change. However, it is possible that detection is hindered because the surprising stimulus is also in a more different spatial location, and therefore not driven by surprise per se but larger shifts in spatial attention. To test this possibility, in experiment 6, participants first experienced alternating stimulus location distributions and orientations. The surprise was defined by a cessation of alternation. In this way, we can model the effect of stimuli broadly alternating or repeating, in location or orientation, alongside the effect of surprise. Experiment 6a performed this manipulation in a paradigm like that employed in experiment 1, and experiment 6b in one like experiment 2. To analyze the relative effects of a change in location alongside a surprising stimulus, experiment 6a was analyzed with the data from experiment 1 and experiment 6b with the data from experiment 2. The data from experiment 6a and 6b are also analyzed alone and presented in Figures S3 and S4, respectively.

#### Experiment 6a

**Figure 6:**
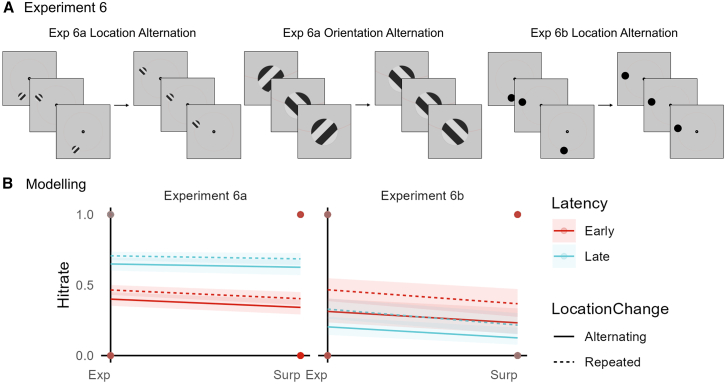
Experiment 6 methods and results (A) In experiment 6a, the trials were similar to those in experiments 1, and in experiment 6b, the trials were similar to those in experiment 2. However, participants experienced first alternating stimulus location distributions and orientations. Surprise was defined by a cessation of alternation. (B) Marginal means of the linear model predicting participant hit rates from the surprise (*x* axis) and alternation of the circumference location (line type) and the latency (color) of the specified detection event. Shaded regions represent the 95% confidence intervals of the fixed-effects model predictions. See also Figures S3 and S4.

The null model (AIC = 14,445.8), with no information regarding surprise, was outperformed by both the surprise model (AIC = 14,438.8) and the interaction model (AIC = 14,439.3). In the surprise model, the surprise of the trial negatively predicted hit rate to the circumference event (log-odds = −0.18, CI = −0.30 to −0.06, *z* = −3.04, *p* < 0.01), as did the latency of the phase shift (log-odds = −0.52, CI = −0.57 to −0.48, *z* = −23.40, *p* < 0.001), and the repetition (log-odds = −0.13, CI = −0.20 to −0.06, *z* = 3.57, *p* < 0.001). Thus, hit rates are higher for expected, late latency, and repeated location trials. The AIC is similar for the surprise and interaction model, but in the latter, the interaction between surprise and latency is not a significant predictor of hit rate (log-odds = −0.08, CI = −0.20–0.04, *z* = −1.23, *p* = 0.217; Figure 6B; Table S5).

#### Experiment 6b

The null model (AIC = 5,141.5), with no information regarding surprise, was outperformed by both the surprise (AIC = 5,130.5) and the interaction model (AIC = 5,132.0). In the surprise model, the surprise of the trial negatively predicted hit rate to the circumference event (log-odds = −0.49, CI = −0.74 to −0.23, *z* = −3.67, *p* < 0.001), the latency of the phase shift positively predicted it (log-odds = 0.30, CI = 0.22–0.38, *z* = 7.73, *p* < 0.001), and repetition was a negative predictor (log-odds = −0.32, CI = −0.45 to −0.19, *z* = 4.96, *p* < 0.001). In the interaction model, there was no significant interaction between the surprise and latency (log-odds = 0.08, CI = −0.14–0.31, *z* = 0.75, *p* = 0.456; see Figure 6C; Table S5).

Experiment 6 allowed us to separate influences of feature repetition and expectation by alternating features trial-to-trial and defining surprise as cessation of alternation. We show that when circumference stimuli are broadly presented at a similar location or orientation, detection of features of those events and events at fixation is facilitated, but this is importantly accompanied by an impairment according to surprise.

## Discussion

The current set of six experiments tested how abrupt changes in environmental regularity—defined either according to spatial location or orientation—alter perception of the surprising event itself and independent incidental events. We were particularly interested in testing the idea that perception is initially biased toward what we expect, but that especially large error signals reactively lead to an increase in sensory gain—to enable high-precision estimates of the environment and accurate model updating.

Consistently across all experiments, participants’ detection of the events decreased after a surprising observation, regardless of the latency of the event and across all event types. The impairment was not restricted to the surprising trial itself and continued for several trials when the change was entirely unanticipated. Experiment 5 suggests that perception of surprising events remains impaired, but recovery from this impairment is faster when changes become more expected as they periodically repeat. The results demonstrate that perception of the expected is indeed generally enhanced but suggest that rather than our particularly hypothesized boost in sensory gain after a highly surprising event, unexpected changes divert resources and impair perception for a few seconds following the surprise. Furthermore, participants show some recovery of this impairment when the surprising events are embedded in an environment with more expected uncertainty.

The findings we present here add to the extant literature demonstrating that expected stimuli are perceived more often, more brightly, and more sharply than their unexpected counterparts.^8^^,^^13^^,^^27^^,^^28^^,^^29^^,^^30^ Our questions are also related to those examined in the attentional capture literature. Attentional capture is a term used to describe phenomena whereby perception is disrupted following events that are attentionally demanding. Given the varied ways in which attention has been defined in the literature,^31^ there are many studies interpreted under this broad umbrella, but most related to our studies are those which examine changes in perception following probabilistically unexpected events. One such set of studies is reported by Asplund and colleagues,^10^ which used an attentional blink paradigm and demonstrated that ongoing target detection was severely impaired after a surprising stimulus. We extend these findings by demonstrating that impairments extend to settings where the surprising events are entirely inconsistent with previously learned variability, being presented only once, i.e., in the situation where they will be most informative for model updating. We also show that they extend across both the surprising events themselves and other simultaneous events, and regardless of latency.

What was unexpected is that a change in environmental regularities systematically reduces detection, regardless of latency and event type, leaving the intriguing question of how observers learn from surprising events when they are perceived so poorly. Indeed, as well as the ideas already outlined, these findings are also inconsistent with the notion that novel stimuli should draw attention and thus be processed more precisely.^32^ We know that participants do learn from environmental changes, at least when the stimulus is detected and discriminable.^5^ It is unclear how this learning is realized in real-world settings where stimuli are not always easily detectable or discriminable, as in this case, and when environments are noisy and attention is diffuse. The proposal that surprising events trigger processes that improve perception would have explained how humans learn from surprising events in the real world, but the fact that we did not observe this here leaves the question still unanswered.

One idea for why detection may improve after an unexpected event is that it triggers noradrenaline release, which may increase sensory gain to improve perception.^4^ While we could not measure noradrenergic processes in the current paradigm, novel stimuli in a range of modalities have been directly shown to elicit locus coeruleus activity in rats and monkeys (see Sara and Bouret^33^), and surprising events similar to those shown here have been shown to evoke pupil dilation in humans,^22^^,^^34^^,^^35^ which is known to covary with noradrenaline release. We therefore assume that our paradigm most likely did elicit noradrenaline release, but that this did not improve detection of hard-to-detect features.

Given that our measures of detection require, of course, an action, it is also worth considering whether response changes following surprise could underlie our effects.^36^ For example, a commonly observed phenomenon is that participants are slower to respond to novel events compared to previously observed events.^37^^,^^38^^,^^39^^,^^40^^,^^41^ However, only in experiment 4, the reaction times were slower for surprising trials compared to expected trials. Second, it is often theorized that these and other motor changes following surprise may be related to reorienting toward the source of surprise^36^ (see also Wessel^42^). However, in experiments 1, 4, and 6a, the detection event is tied to the surprising stimulus itself. Thus, a re-orienting mechanism would arguably provide a benefit, rather than a decrement, to performance, even if not immediately but at the later delays we examined. Of course, this was not found. We speculate that we may not have found these effects because all events were designed to span a small visual angle to discourage shifts of spatial attention, but it would be informative to interrogate these interdependences further in future, e.g., neural, work.

Our design was inspired by previous work that established expectations through presenting consecutive stimuli around a fixed spatial location and inducing surprise by changing this location.^22^^,^^23^ The design was partly motivated by the possibility that selection processes reactively enhancing the perception of surprising events may be driven by shifts in spatial attention. Interestingly, however, we found qualitatively similar effects regardless of whether the surprise was generated by changing the stimulus location or by changing the stimulus orientation at the same location. Experiment 6 took inspiration from Lee and Rideaux^17^ to further show, by rendering a repeat in location unexpected, that while impairments due to shifts in location may contribute to our effects, there are dissociable influences of statistical surprise. Therefore, environmental changes need not invoke shifts in spatial attention to impair perception (also in line with Asplund et al.^10^). Relatedly, the mechanisms underlying repetition suppression phenomena are still not well understood,^43^^,^^44^^,^^45^ but experiment 6 dissociates repetition from expectation, and shows that surprise-induced impairments are not entirely attributable to repetition.

While we know that that surprising events can attract overt attention,^46^^,^^47^^,^^48^^,^^49^^,^^50^ we intentionally discouraged overt shifts by designing the entire display to be parafoveal and assigning a difficult task at the center. Participants completed practice rounds for both the circumference and the central tasks, which were difficult and even more difficult if not obeying instructions to stay fixated on the central event. We could not monitor fixation in this online task, opting to collect thousands of participants to characterize behavioral profiles at the expense of eye-tracking data. Future research could employ a lab setup with eye tracking to determine how fixation change may mediate some of this nuanced learning-perception relationship. However, it would seem unlikely that changes in fixation entirely drive effects when the same results were obtained with orientation changes in experiment 1.

Here, events were all presented within central vision in a simple environment. One may therefore propose that there is no need for additional orienting or foraging processes in this simple visual context, as in otherwise observed “epistemic foraging” responses to surprise.^51^^,^^52^^,^^53^^,^^54^ It is possible that a surprising event in noisier, real-world settings with harder-to-detect and harder-to-discriminate stimuli would in fact trigger participants to explore their environments and forage for information to build a richer percept and world model. Alternatively, even in a simple environment, surprising events may be encoded especially well in some layers of early cortex,^55^ and such encoding may enable model updating automatically even in the absence of a strong percept.

It is also possible that surprising events of the kind we presented in this paradigm do not increase sensory gain because model updating is only of the location or orientation parameter. It has been argued that this type of learning is qualitatively different from so-called structure learning, in which there is a change in the latent causes underlying the observed states.^56^ To return to our kettle example, if I see the kettle in a new location on my kitchen surface, I already know there can be some variability on this dimension. If, however, I see the kettle floating in mid-air, I would arguably add a new variable of interest to my mental model, perhaps related to self-propulsion or susceptibility to gravity. Adding this new variable to the model is one of a collection of restructuring operations,^57^ and it is possible that only this latter type of learning increases sensory gain. Future research should investigate this question with a paradigm that allows for a change in latent cause without fundamentally changing the task.^56^

In conclusion, when environmental regularities change, we present evidence that detection of features of the surprising events themselves, and other events in the environment, is impaired. This impairment is relative to events consistent with learned uncertainty and persists for at least a few seconds following the surprise, though when changes become more frequent recovery is immediate. These findings suggest that perception of the expected is generally enhanced and that surprising observations do not reactively improve perception across channels. We have outlined a range of possibilities for how we may therefore update our models accurately when the world changes in spite of poorer perception.

### Limitations of the study

An interesting addition to the current studies would be concurrent eye tracking. Despite designing the study to maximize the motivation to fixate on the central point, we cannot rule out occasional saccades and their impact on detection. Moreover, pupillometry would enable us to examine more readily the role of catecholaminergic processes in detection.

## Resource availability

### Lead contact

Further information and requests for resources should be directed to and will be fulfilled by the lead contact, Nick Simpson (nicholas.simpson.23@ucl.ac.uk).

### Materials availability

No new materials have been generated.

### Data and code availability

• Anonymized data have been deposited on OSF: https://osf.io/j867v/. They are publicly available as of the date of publication.
• All original code has been deposited on OSF: https://osf.io/j867v and is publicly available as of the date of publication.
• A pre-print is available on OSF: https://doi.org/10.31234/osf.io/xsn7v. We pre-registered the initial design and hypotheses for this set of studies before data were collected, and the pre-registration can be found on OSF: https://osf.io/2mdtr, and for experiment 6, on OSF: https://osf.io/bx4us https://osf.io/mt5n8. Minimal experiment code has been made for presentation purposes and is available on Github: https://github.com/nick-c-simpson/SurpriseImpairsPerceptionGifs.git.
• Any additional information required to reanalyze the data reported in this paper are available from the lead contact upon request.

## Acknowledgments

This work was funded by a 10.13039/501100000781European Research Council (ERC) consolidator grant (101001592) under the European Union’s Horizon 2020 research and innovation programme and 10.13039/501100000275Leverhulme Trust project grant (RPG-2022-358), both awarded to C.P. We are grateful to the two anonymous reviewers for their helpful feedback, most notably suggesting the addition of experiment 6, as well as past and present members of the Action and Perception Lab for useful input throughout the studies.

## Author contributions

E.K.W. contributed to conceptualization, data curation, formal analysis, investigation, methodology, project administration, software, validation, visualization, writing – original draft, and writing – review and editing. N.S. contributed to data curation, formal analysis, investigation, methodology, software, validation, visualization, and writing – review and editing. C.P. contributed to conceptualization, funding acquisition, methodology, project administration, resources, supervision, and writing – review and editing.

## Declaration of interests

The authors declare no competing interests.

## STAR★Methods

### Key resources table

**Table:** 

REAGENT or RESOURCE	SOURCE	IDENTIFIER
**Deposited data**		

Anonymised Data	This Paper	OSF: https://osf.io/j867v/

**Software and algorithms**		

Analysis Code	This Paper	OSF: https://osf.io/j867v
Minimal Stimulus Code	This Paper	Github: https://github.com/nick-c-simpson/SurpriseImpairsPerceptionGifs.git

### Experimental model and study participant details

#### Experiment 1

Sample size was fixed at 100 participants per between-participants manipulation. We were not aware of comparable studies upon which to base a power analysis so selected a sample size to maximise power while keeping the study costs reasonable. Exclusion criteria were set before recruitment, such that participants were only invited to participate if they had previously reported that they were fluent in English, had normal or corrected-to-normal vision, were not colour blind, were not autistic (due to planned follow-up work) and had a Prolific approval rate of more than 95%. This study was approved by the local ethics committee, and participants received a small amount of money, approximately equivalent to £8 per hour, as a thank you for taking part.

619 participants (241 reported their sex as female, 359 as male, 19 preferred not to choose one of those options) were recruited online using Prolific.^58^ There is no reason to think that these mechanisms underlying visual processing and statistical learning studied throughout Experiments 1-6 would differ according to sex and therefore we did not examine its influence. Participants were removed from further analysis if they quit the experiment before completion, leaving 597 participants (mean age = 30 years, SD = 10). Participants were randomly assigned to early latency groups, late latency groups, or false alarm groups. Within these groups participants were split such that half of participants were presented first with a location change and second with an orientation change, and the other half experienced the other order.

#### Experiment 2

277 participants were recruited online using Prolific,^58^ 209 of whom contributed a dataset (101 female, 105 male, 3 preferred not to choose one of those options). Participants were removed from further analysis if they did not complete the experiment, leaving 190 individuals included in the final analysis (mean age = 34 years, SD = 13). All participants saw central events at 0 ms and 200 ms latencies during run 1 and were randomly assigned to see central events at 0 ms latency (N=105) or at 200 ms latency (N=85) on the critical trials.

#### Experiment 3

248 participants were recruited online using Prolific,^58^ 208 of whom contributed a dataset (84 female, 119 male, 5 preferred not to choose one of those options). Participants were removed from further analysis if they did not complete the experiment, leaving 191 individuals (mean age = 29 years, SD = 9). All participants saw central events at 0 ms and 200 ms latencies during run 1, and were randomly assigned to see central events at 0 ms latency (N=94) or at 200 ms latency (N=97) on the critical trials.

#### Experiment 4

239 participants were recruited online using Prolific,^58^ 212 of whom contributed a dataset (85 female, 115 male, 12 preferred not to choose one of those options). Participants were removed from further analysis if they did not complete the experiment, leaving 194 individuals (mean age = 33 years, SD = 13). All participants saw oddball circumference events at expected locations during run 1 and were randomly assigned to see an oddball circumference event at the expected location (N=91) or at the unexpected location (N=103) on the critical trials.

#### Experiment 5

The sample size was fixed at 200 participants for Experiment 5, as the manipulation in this experiment was entirely within-participant. 201 participants (126 female, 74 male, 1 preferred not to choose one of those options) were recruited online using Prolific.^58^ Participants were removed from further analysis if they quit the experiment before completion, leaving 198 participants (mean age = 35 years, SD = 11).

#### Experiment 6

*Experiment 6a.* 218 participants were recruited online using Prolific^58^ (137 female, 81 male). Participants were removed from further analysis if they did not complete the full study, leaving 200 included participants (mean age = 37.4 years, SD = 9.7). Data from Experiment 1 were also included in the analysis.

*Experiment 6b.* 205 participants were recruited online using Prolific^58^ (133 female, 72 male). Participants were removed from further analysis if they did not complete the full study, leaving 199 included participants (mean age = 40.4 years, SD = 10.4). Data from Experiment 2 were also included in the analysis.

### Method details

#### Experiment 1

*Apparatus.* All experiment code was written in Javascript and jsPsych^59^ (de Leeuw, 2015) using JATOS and hosted on MindProbe.^60^ Participants took part remotely on their own devices, and we restricted participation to computers with a physical keyboard, such that the whole display was visible and participants could maintain posture easily with hands on the response keys.

*Stimuli and procedure.* The display consisted of a grey background (RGB 200, 200, 200) with a large red circle with radius 100 pixels, which subtended ∼4-5 degrees of visual angle when viewed at 50-60 cm, centred on a small ring-shaped fixation point in the centre of the screen. This fixation point consisted of a black-and-white target with a radius of 8 pixels (see Figure 1A; and https://github.com/nick-c-simpson/SurpriseImpairsPerceptionGifs.git Experiment 1).

The circumference stimuli consisted of black and white square-wave gratings of radius 20 pixels, presented for 1000 ms. The circumference stimuli were static on around 75% of trials, while on oddball trials there was a small phase shift for 50 ms. This phase shift occurred either 50 ms or 300 ms after stimulus onset. On a further ∼25% of trials, in addition to the standard circumference stimulus, a thin grey ring (RGB 190, 190, 190) was presented around the fixation point for 50 ms. These are referred to as the central events. This event constituted a width of 1 pixel and a radius drawn randomly from a uniform distribution between 20-40 pixels, centred on the fixation point. There was a jittered inter-stimulus interval of 200-700 ms between the presentation of the circumference stimuli.

Participants’ task was to maintain fixation on the central point, to press J when they detected the oddball circumference stimulus and F when they detected the central event. Participants were first given a block of 20 trials to practise the central task in isolation, during which the central event appeared 6 times. Participants could proceed to the next block only once they had reported the correct number of central events ±1, that is, allowing for a maximum of one miss or false alarm. They subsequently undertook a block of 20 trials to practise the circumference task in isolation, which contained 6 oddball circumference stimuli, and again were only allowed to proceed when their performance reached the ±1 threshold. They were finally presented with the main task and asked to respond to both types of event.

Circumference stimuli during practice trials were presented at locations drawn from a normal distribution centred around a fixed mean, truncated at ±30 degrees, on the circumference of the red circle. During the main task, circumference stimuli were initially presented at locations drawn from the same distribution, until the mean of the underlying distribution was changed abruptly and without warning (location change). Circumference stimuli were also initially presented with an orientation of 45° until the orientation was changed abruptly and without warning to 135° (orientation change). The order of the changes in location and orientation was counterbalanced.

The main task consisted of 3 runs, the first two of which were 32 trials long, and the third only 2 trials. The second and third runs each contained a change on one dimension – stimulus location or stimulus orientation – with the order counterbalanced across participants. The final run only lasted for 2 trials as this was deemed sufficient for measuring the change in detection following an unexpected event. During the main task, for the 32 trials in run 1, circumference stimuli were presented at locations drawn from the same spatial distribution and at the same grating orientation. Run 2 presented a change in either the location or the orientation of the circumference stimuli, which was subsequently held constant for the run. Finally, at the start of run 3, participants who had been presented with a location switch in run 2 were presented with an orientation switch, and vice versa.

*Changepoint probability.* We used changepoint probability modelling^24^^,^^25^^,^^26^ to quantify the amount of surprise for each presented stimulus depending on the location of the circumference stimulus and the orientation of the grating, and used this to predict hitrates for the two perceptual tasks. It is worth noting that, given our task structure, these changepoints fairly reliably followed the coded shift in distribution.

*Surprise-induced weighting factor.* To compute the SIWF, we identified all trials with changepoint probability over a certain threshold (either a value of 0.9 or the 99.9^th^ percentile changepoint probability that participant had seen, whichever was lower) and assigned a weighting factor by sampling the beta-distribution (see Figure 2C) at evenly-spaced intervals for the trials following a changepoint. Therefore, in contrast with the above analysis, the surprise-induced weighting factor is modelled as triggered by a surprising event and decaying gradually over the following trials, rather than elicited only for trials that differ from the previously-observed distribution (see Figures 2A and 2B). Additionally, the surprise-induced weighting factor remains at zero until a significantly large changepoint probability is observed. Thus this analysis removes the effect of the smaller, but more frequent, changepoint probabilities on hitrate that occur due to minor differences in true location drawn from the same mean location. However, of note, difference in results between the changepoint probability and surprise-induced weighting factor appear driven by the more gradual decay. This is demonstrated by allowing the SIWF to gradually decay, spike (high weight on trial 1, then immediately return to 0), or step (high weight on all trials following a high change point) and we find that the spike model generally provided a poorer fit (see Table S1).

#### Experiment 2

Stimuli and procedure were identical to Experiment 1 except that the circumference stimuli consisted of solid-coloured dots of radius 20 pixels, presented for 150 ms (See Figure 3A and https://github.com/nick-c-simpson/SurpriseImpairsPerceptionGifs.git Experiment 2). The circumference stimuli were black on around 75% of trials, while on oddball trials they were grey (RGB 120, 120, 120). In Experiment 2 the event of interest was the central event, which lasted 50 ms with onset either 0 ms or 200 ms after the onset of the circumference stimulus.

During the main task, for 45 trials in run 1, circumference stimuli were presented at locations drawn from the same distribution. At the start of run 2, there was a change in location displacing the circumference stimulus 90° clockwise or anti-clockwise from the location of the most recent previous stimulus. This location was held constant for both trials of run 2. On both trials in run 2, we presented a central event requiring a response.

#### Experiment 3

Stimuli and procedure were identical to those of Experiment 2, with the exception of the size of the displacement of the circumference stimulus location, which in this case was 180 degrees from the location of the most recent previous stimulus. This location was held constant for both trials of run 2. On both trials of run 2, we presented a central event requiring a response.

#### Experiment 4

Stimuli and procedure were identical to Experiment 2 except that on each trial participants saw two circumference stimuli presented simultaneously (See Figure 4A and https://github.com/nick-c-simpson/SurpriseImpairsPerceptionGifs.git Experiment 4). The circumference stimuli were both black on around 75% of trials, while on oddball trials, one of the circumference stimuli was grey (RGB 120, 120, 120).

During the main task, for the 45 trials in run 1, one circumference stimulus was presented at locations drawn from the same distribution, while the other was presented at a location offset from the first by 20-40° either clockwise or anti-clockwise. In run 1, all oddball circumference stimuli were shown at the location drawn from the original distribution, leading to comparable spread in the locations of the oddball stimuli as in Experiments 1, 2, and 3 despite the slightly larger spread of locations of circumference stimuli overall.

At the start of run 2, there was a switch in location of one of the presented circumference stimuli by 90° from the last previous location, while the other was presented at an expected location. On both trials in run 2, we presented one grey circumference stimulus, requiring a response, and one black circumference stimulus. In these final two trials, one group of participants saw the grey circumference stimulus at the expected location and the other group saw the grey circumference stimulus at the unexpected location. The changepoint probability was defined for the stimulus that was grey on these final two trials. The raw hitrates for the two experimental groups are presented in Figure S2, however the groups are combined for the main task analysis.

#### Experiment 5

The circumference stimuli consisted of circles of radius 20 pixels, presented for 150 ms. The circumference stimuli were black on around 75% of trials, while on oddball trials they were grey (RGB 120, 120, 120). There was a fixed inter-stimulus interval of 850ms between the presentation of the circumference stimuli. On a further ∼25% of trials, there was a grey ring presented around the centre of the display for a duration of 50ms. The onset of the central event was presented at different latencies relative to the onset of the circumference stimulus, from 0 ms to 300 ms in increments of 50 ms. The greater range of latencies allowed us to determine whether there were any short-lived effects missed with the designs of Experiments 1-4.

In Experiment 5, the circumference stimuli during practice trials were presented at locations drawn from a uniform distribution spanning 0-360°, meaning they could appear anywhere on the red circle. During the main task, circumference stimuli were presented at locations drawn from a normal distribution around one of the cardinal points, and this location distribution was changed every 10-20 trials for a total of 32 runs, to examine the perceptual consequences of changes that become expected (O’Reilly et al., 2013). A pseudorandomisation ensured that a central event was presented on the first trial of a new run once for each latency, leading to 7 out of 32 runs with a central event presented on the first trial.

#### Experiment 6

Experiment 6a presented stimuli like in Experiment 1, where grating stimuli appeared on the circumference and participants were required to detect a phase shift at 50 and 300 ms. However, now circumference stimuli alternated between two location distributions and orientations trial-to-trial. The first surprise, at the start of run 2, pertained to the location repeating rather than alternating, and the second, at the start of run 3, to the orientation repeating. For each participant, an odd-trial and an even-trial mean location for the circumference stimulus was selected from the cardinal axes, ensuring that the difference between the locations was 90 degrees. The circumference stimulus location was drawn from the two means on alternating trials such that participants could learn to expect that the location should change.

During the practice and the first run of 45 trials, the participants could build the expectation of alternation. Due to the expectation of alternation, changepoint modelling could not be used to model participant behaviour. Thus, to analyse these data we used a model free approach whereby the first two trials after the change in statistical structure were labelled as ‘Surprising’ and all other circumference trials were labelled as ‘Expected’. For this analysis, data were combined with data from Experiment 1. Experiment 6b performed the same alternation manipulation but now as a variant of Experiment 2. I.e., the circumference events were grey circles rather than gratings, and we manipulated the timing of events at fixation - 0 ms or 200 ms with respect to the circumference events. Specifically, the circumference locations broadly alternated rather than repeated, and the surprise at the start of run 2 pertained to the location repeating. For this analysis, data were combined with those from Experiment 2. The data for the alternation studies is presented in isolation in Figures S3 and S4.

### Quantification and statistical analyses

#### Experiment 1

*Analysis software.* All responses were recorded in a json object stored in MindProbe and processed in R version 4.2.2^61^ and RStudio version 2023.03.0+386.^62^ The data were read and cleaned using the R packages jsonlite^63^ and reshape2.^64^ Changepoint modelling was conducted with bcp.^24^^,^^25^^,^^26^ Multi-level models were run with lme4.^65^ Plots were made with ggplot2,^66^ effects^67^^,^^68^ and sjPlot,^69^ and regression tables with sjPlot.^69^ Degrees of freedom are calculated using the Wald method and predictors in multi-level models were considered as significant if the z-value exceeded ±1.96.

*Analysis Procedure.* Responses were assigned to the trial in which they occurred, and reaction times computed between the onset of the stimulus event and the key press. Responses were included in further analysis if they were made more than 100 ms and no more than 800 ms after the onset of the relevant stimulus event. The locations of circumference stimuli were recoded as the shortest distance around the circle from the generative mean of the first run of trials (using code adapted from Vaghi et al.^23^), to correct for the circular nature of the stimuli and to render locations comparable across participants. We analysed participants’ hits to the oddball circumference stimuli (coded as 0 for a miss and 1 for a hit) using binomial generalised linear multi-level models, to accommodate the binary outcome. See Model Specification for the full details. The false alarm group are analysed separately in Figure S1.

*Reaction Time Analysis.* To test whether participant reaction times were different in response to surprising trials compared to expected trials, the first two trials after a surprise were defined as surprising. The reaction times (RT) were then predicted from the trial expectation, detection event latency, and trial number using three linear models:

$${Interaction}_{model}=\log\left(RT\right)∼Expectation*Latency+trialNumber+\left(1+trialNumber|PID\right)$$

$${Expectation}_{model}=\log\left(RT\right)∼Expectation+Latency+trialNumber+\left(1+trialNumber|PID\right)$$

$${Null}_{model}=\log\left(RT\right)∼Latency+trialNumber+\left(1+trialNumber|PID\right)$$

The results of the RT analysis for all experiments are presented in Figure S5.

#### Model specification

Models were run with sum-to-zero coding such that all effects are estimated at the average value of other predictors, with a hypothesis-led approach to model building, using the Akaike Information Criterion (AIC) for model comparison and selection. We applied the recommendation that an AIC value more than 3 below another indicates a better-fitting model, and subsequently determined the significance of predictors from the winning model. The trial number predictors were all z-scored to place all values at a similar scale. We fit models with a maximal random effect structure without causing the model to be singular or have a degenerate Hessian. However, all conclusions remain the same when including only a random slope for trial number. The syntax of the generalised multi-level models was as follows, using a binomial error distribution and logit link implemented with glmer:

modelC<−glmer(A∼B,data=experimentX,family=binomial)

Below is shown the equation A∼B for each model fit. The abbreviations used are:

CPP = changepoint probability

SIWF = surprise induced weighting factor

TN = trial number

PID = participant ID

PS = phase shift

cen = central

$$\;\text{model}1_{\text{CPP}}=\text{hitCircumference}∼{\text{CPP}}_{\text{location}}*\text{Latenc}y_{\text{PS}}+{\text{CPP}}_{\text{orientation}}*\text{Latenc}y_{\text{PS}}+\text{TN}$$

$$+\left(1+\text{TN}+{\text{CPP}}_{\text{location}}|\text{PID}\right)$$

$$\;\text{model}1_{\text{SIWF}}=\text{hitCircumference}∼\text{SIWF}*\text{Latenc}y_{\text{PS}}+\text{TN}+\left(1+\text{TN}+\text{SIWF}|\text{PID}\right)$$

#### Experiment 2

$$\;\text{model}2_{\text{CPP}}=\text{hitCentral}∼\text{CPP}*\text{Latenc}y_{\text{cen}}+\text{TN}+\left(1+\text{CPP}+\text{Latency}|\text{PID}\right)$$

$$\;\text{model}2_{\text{SIWF}}=\text{hitCentral}∼\text{SIWF}*\text{Latenc}y_{\text{cen}}+\text{TN}+\left(1+\text{SIWF}+\text{Latency}|\text{PID}\right)$$

#### Experiment 3

$$\;\text{model}3_{\text{CPP}}=\text{hitCentral}∼\text{CPP}*\text{Latenc}y_{\text{cen}}+\text{TN}+\left(1+\text{CPP}+\text{Latency}|\text{PID}\right)$$

$$\;\text{model}3_{\text{SIWF}}=\text{hitCentral}∼\text{SIWF}*\text{Latenc}y_{\text{cen}}+\text{TN}+\left(1+\text{SIWF}+\text{Latency}|\text{PID}\right)$$

#### Experiment 4

$$\;\text{model}4_{\text{CPP}}=\text{hitCentral}∼\text{CPP}+\text{TN}+\left(1+\text{CPP}+\text{TN}|\text{PID}\right)$$

$$\;\text{model}4_{\text{SIWF}}=\text{hitCentral}∼\text{SIWF}+\text{TN}+\left(1+\text{SIWF}+\text{TN}|\text{PID}\right)$$

#### Experiment 5

$$\;\text{model}5_{\text{CPPNoInt}}=\text{hitCentral}∼\text{CPP}*\text{Latenc}y_{\text{cen}}+\text{CPP}+\text{normalisedRunNumber}+\left(1|\text{PID}\right)$$

$$\;\text{model}5_{\text{CPP}}=\text{hitCentral}∼\text{CPP}*\text{Latenc}y_{\text{cen}}+\text{CPP}*\text{normalisedRunNumber}+\left(1|\text{PID}\right)$$

$$\;\text{model}5_{\text{SIWFNoInt}}=\text{hitCentral}∼\text{SIWF}*\text{Latenc}y_{\text{cen}}+\text{SIWF}+\text{normalisedRunNumber}+\left(1|\text{PID}\right)$$

$$\;\text{model}5_{\text{SIWF}}=\text{hitCentral}∼\text{SIWF}*\text{Latenc}y_{\text{cen}}+\text{SIWF}*\text{normalisedRunNumber}+\left(1|\text{PID}\right)$$

#### Experiment 6

Changepoint modelling is not possible for alternating expectations, hence, in Experiment 6 the two trials immediately following a change in environmental statistics were defined as surprising and all other trials defined as expected. The hitrate was modelled depending on the Experiment in which the data were collected (expecting repeated locations or expecting alternating locations), whether the trial was Alternating (either in the same or a different location to the previous trial), the Latency, the Trial Number, and whether the trial was Surprising.

$$\;\text{model}6_{\text{null}}=\text{hit}∼\text{TN}+\text{Experiment}+\text{Alternating}+\text{Latency}+\left(1+\text{TN}|\text{PID}\right)$$

$$\;\text{model}6_{\text{surprise}}=\text{hit}∼\text{TN}+\text{Experiment}+\text{Alternating}+\text{Latency}+\text{Surprise}+\left(1+\text{TN}|\text{PID}\right)$$

$$\;\text{model}6_{\text{interaction}}=\text{hit}∼\text{TN}+\text{Experiment}+\text{Alternating}+\text{Latency}*\text{Surprise}+\left(1+\text{TN}|\text{PID}\right)$$
