# Supplementary material for: Violating statistical structure impairs detection of deviant and incidental events
Source: iScience. 2026 Jun 17;29(7):116383. doi: 10.1016/j.isci.2026.116383 (PMC13293695; doi:10.1016/j.isci.2026.116383)
Supplement: Document S1. Figures S1–S5 and Tables S1–S5 [file mmc1.pdf]

iScience, Volume *29*

Supplemental information

Violating statistical structure impairs detection of deviant and incidental events

Emma K. Ward, Nick Simpson, and Clare Press

Supplemental Figure 1. Marginal means of the false alarm rate predicted by SIWF in Experiment 1.

In Experiment 1, a group of participants were presented with no detection event on the two trials post-surprise. These data were analysed by predicting whether there was a false alarm depending on the trial number, the changepoint probability (CPP), or the surprise-induced weighting factor (SIWF). A SIWF model (AIC = 1882.8) was found equivalent to a null model (AIC 1883.7) and both outperformed a CPP model (AIC = 1887.6). In the SIWF model, the SIWF was not a significant predictor of false alarm (log-Odds = -0.60, CI = -1.36 - 0.16, z = -1.54, p = 0.12). The marginal means from the SIWF model are plotted.

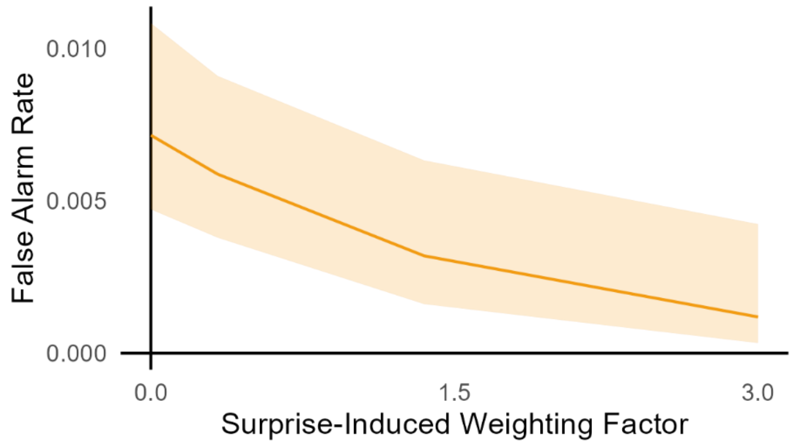

Supplemental Figure 2. Raw hitrates of the expected and surprising location groups in Experiment 4.

The Expected group saw detection events on the circumference stimulus that remained in the expected location and the Surprising group saw detection events on the stimulus that moved to a new location. The x-axis separates the trial types. 'Surprise' trials are defined as the first two trials after one of the stimuli moves location and all other trials are 'Expected'. There is no change in hitrate for the Expected group on surprising trials, but there is for the Surprising group. One might wonder why there is no decrement in the Expected group, given influences of surprise on detection of events at fixation. While these experiments were not designed to analyse this feature, we speculate that the null effect here is that a pre-activation of expected stimulus features which facilitates performance (Press et al., 2020) may be combined with the decrement caused by surprise elsewhere in the display.

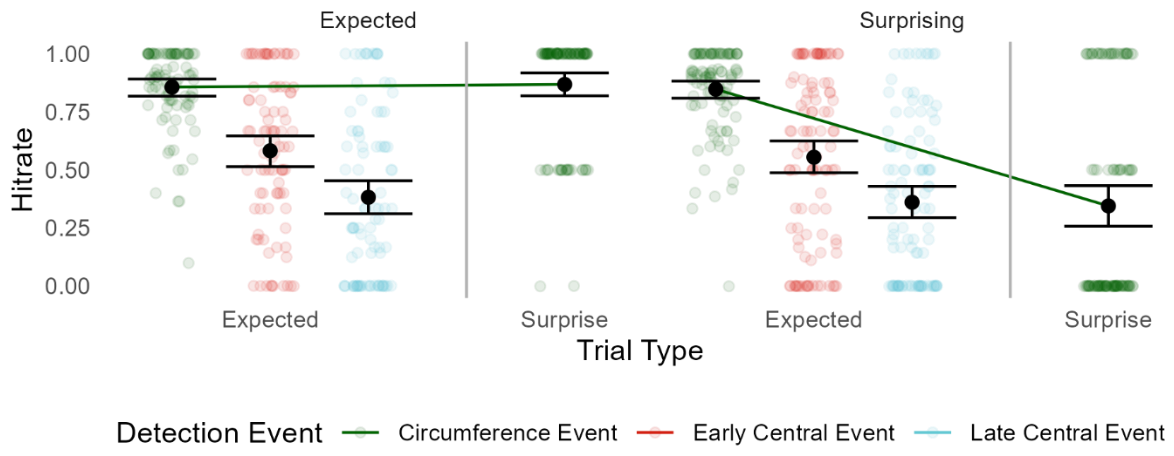

Supplemental Figure 3. Experiment 6a Results.

A In Experiment 6a, the trials were similar to those in Experiment 1, but participants were initially exposed to alternating stimulus distributions. Surprise was defined by a cessation of alternation. **B** Raw hitrates of expected trials (pre-surprise) and surprise trials (first two after surprise) for circumference, early central and late central events, separately for the location and orientation surprise events. **C** Marginal means of the surprise model with no interaction with detection event latency. **D** Marginal means of the model allowing the surprise to interact with the latency of detection events (0 ms in red and 200 ms in blue). As we would expect there to be a detection advantage for the stimulus remaining in the same place as the previous trial, in the Main Text analysis we account for both the advantage of trials remaining in the same place and the advantage of trials being in an expected location. Therefore the null result when analysing solely Experiment 6a suggests a disadvantage of equal magnitude for changing and being statistically surprising.

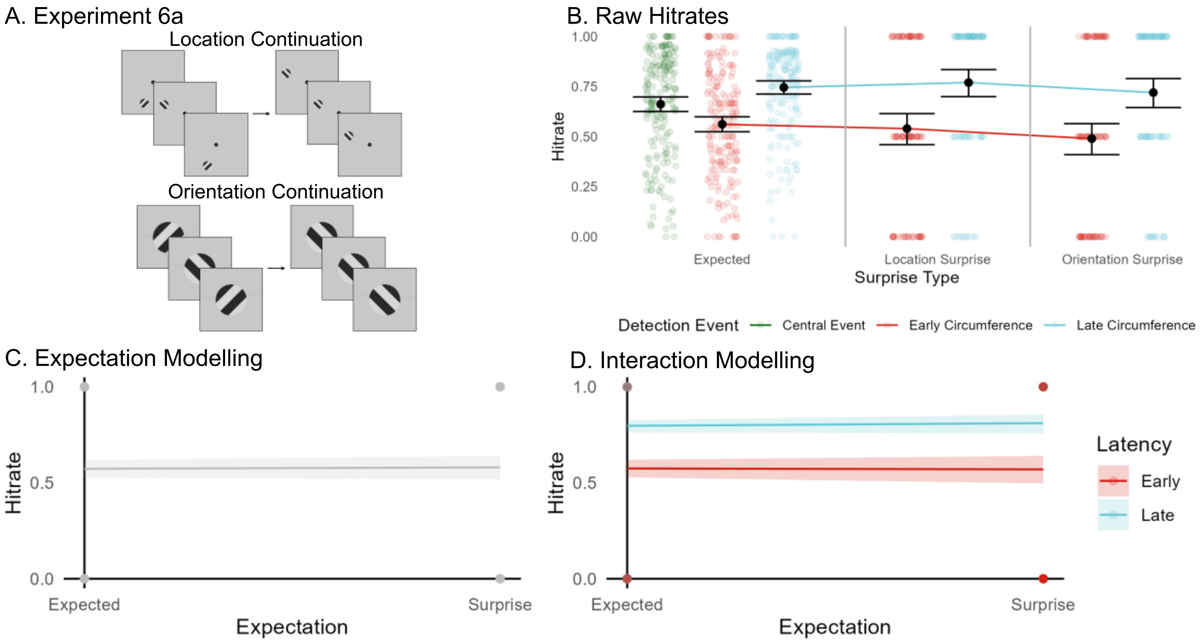

Supplemental Figure 4. Experiment 6b Results.

A In Experiment 6b, the trials were similar to those in Experiment 2, but participants were initially exposed to alternating stimulus distributions. Surprise was defined by a cessation of alternation. **B** Raw hitrates of expected trials (pre-surprise) and surprise trials (first two after surprise) for circumference, early central and late central events. **C** Marginal means of the surprise model with no interaction with detection event latency. **D** Marginal means of the model allowing the surprise to interact with the latency of detection events (0 ms in red and 200 ms in blue). The increase in hitrate for Surprise compared to Expected trials suggests that the decrement associated with switching location is larger than that associated with surprise.

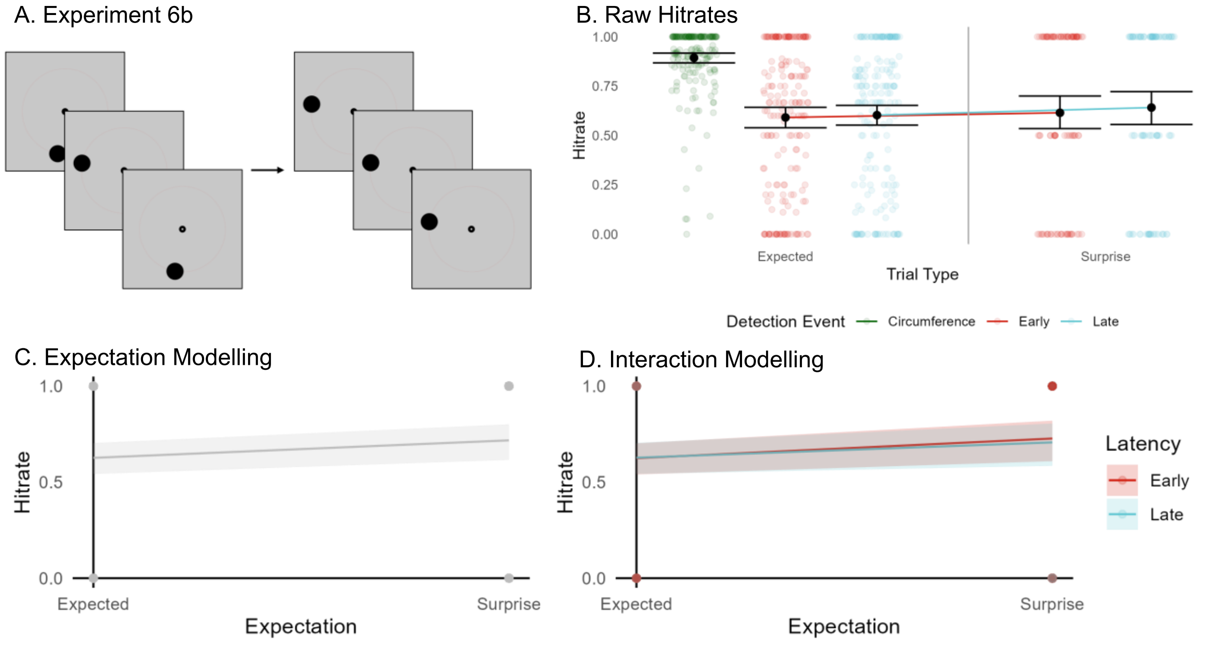

Supplemental Figure 5. Reaction Time Analysis Across Experiments 1-6

Marginal means from multi-level models predicting participant reaction times (log(RT)) from expectation and detection event latency. Only in Experiment 4 was there a difference in RT depending on expectation, where Surprising trials had slower RTs compared to Expected trials. For all other Experiments, the null model explained the data best.

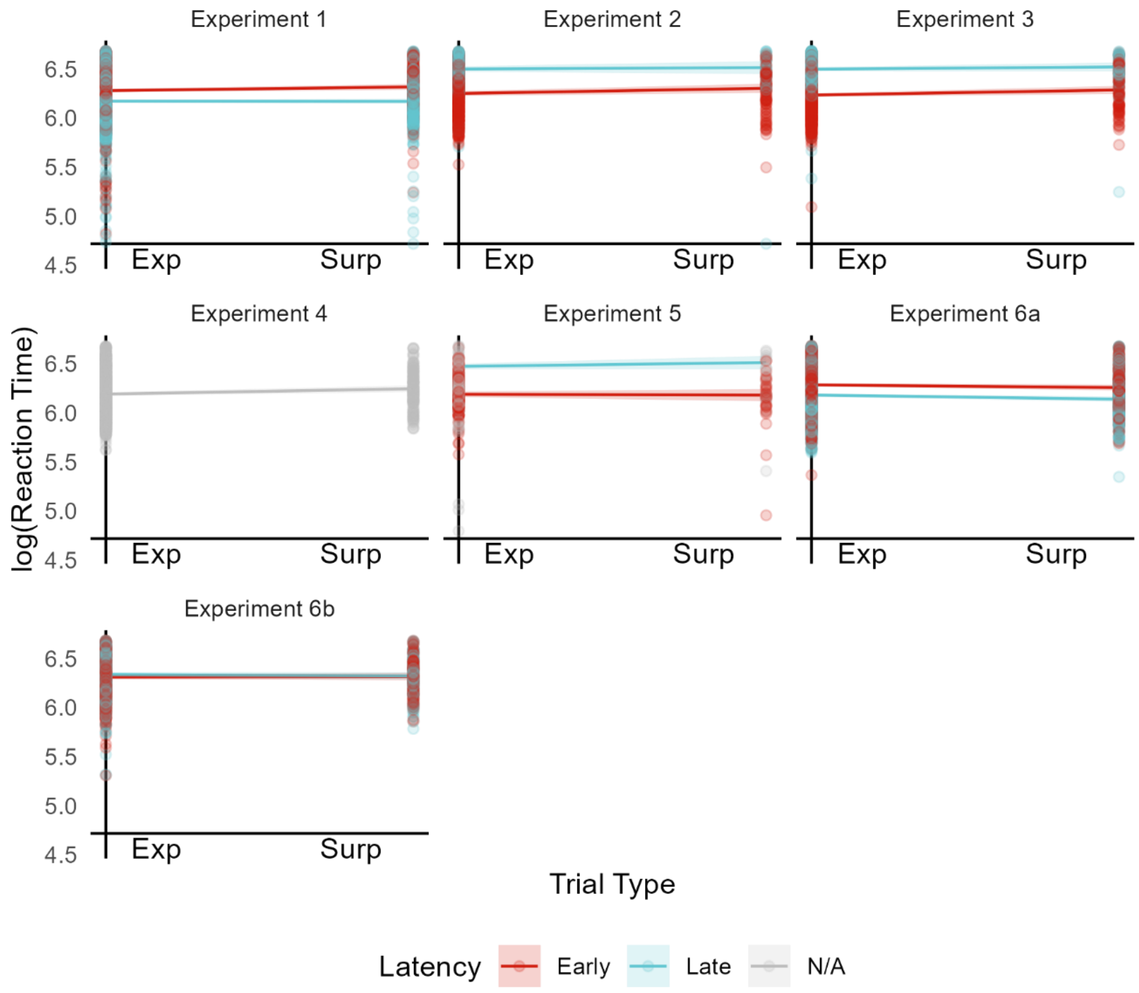

Supplemental Table 1. Comparing the model fits of possible weighting shapes.

Following a trial with a large changepoint probability (CPP), the change in hitrate was modelled with a gradual decay described for the surprise-induced weighting factor (SIWF), a spike (high for one trial and immediately return to zero), or a step (remain high). Only for Experiment 5 was did the spike model provide a better fit than the SIWF or Step models.

	CPP	SIWF	Spike	Step
Experiment 1	**8324.8**	8320.9	8330.8	8325.0
Experiment 2	2540.5	2524.6	2540.3	**2514.6**
Experiment 3	2831.1	**2817.7**	2831.9	2825.7
Experiment 4	2109.2	1993.3	2094.0	**1984.5**
Experiment 5	27982.9	27982.1	**27980.8**	27990.0

Supplemental Table 2. Changepoint model outputs for Experiment 1-4.

	**ST2 Experiment 1 - Changepoint**		
Predictors	Log-Odds	CI	Statistic
Intercept	0.40 ***	0.26 – 0.54	5.72
CPP Location	-0.56 ***	-0.83 – -0.30	-4.14
Latency	-0.51 ***	-0.57 – -0.45	-16.65
CPP Orientation	-0.30 *	-0.55 – -0.04	-2.28
Trial Number	-0.37 ***	-0.44 – -0.30	-10.59
CPP Location x Latency	-0.21	-0.48 – 0.06	-1.53
CPP Orientation x Latency	0.07	-0.18 – 0.33	0.57
Random Effects			
σ^2	3.29		
$\tau_{00,P}$	1.37		
$\tau_{11,P.curTrial}$	0.11		
$\tau_{11,P.pCP}$	0.18		
ρ_{01}	0.05		
	-0.85		
ICC	0.31		
N_P	352		
Observations		7040	
Marginal R^2/Conditional R^2		0.087 / 0.368	
	p<0.05 ** p<0.01 *** p<0.001		

	**ST2 Experiment 2 - Changepoint**		
Predictors	Log-Odds	CI	Statistic
Intercept	-0.52 **	-0.83 – -0.20	-3.22
CPP	-0.81 **	-1.37 – -0.26	-2.86
Latency	0.57 ***	0.45 – 0.69	9.01
Trial Number	-0.14 *	-0.25 – -0.02	-2.29
CPP x Latency	0.42	-0.07 – 0.91	1.69
Random Effects			
σ^2	3.29		
$\tau_{00,P}$	4.03		
$\tau_{11,P.Latency}$	0.04		
$\tau_{11,P.pCP}$	0.01		
ρ_{01}	0.02		
	0.50		
ICC	0.55		
N_P	191		
Observations		2424	
Marginal R^2/Conditional R^2		0.057 / 0.579	
	p<0.05 ** p<0.01 *** p<0.001		

	**ST2 Experiment 3 - Changepoint**		
Predictors	Log-Odds	CI	Statistic
Intercept	0.44 ***	0.20 – 0.67	3.64
CPP	-1.30 ***	-1.73 – -0.87	-5.90
Latency	0.39 ***	0.27 – 0.50	6.70
Trial Number	-0.05	-0.16 – 0.06	-0.89
CPP x Latency	0.02	-0.37 – 0.40	0.08
Random Effects			
σ^2	3.29		
$\tau_{00,P}$	2.15		
$\tau_{11,P.Latency}$	0.06		
$\tau_{11,P.pCP}$	0.11		
ρ_{01}	-0.25		
	-0.61		
ICC	0.40		
N_P	191		
Observations		2433	
Marginal R^2/Conditional R^2		0.050/0.428	
	p<0.05 ** p<0.01 *** p<0.001		

Supplemental Table 3. SIWF model outputs for Experiment 1-4.

			ST2 Experiment 4 - Changepoint
Predictors	Log-Odds	CI	Statistic
Intercept	2.10 ***	1.87 – 2.33	17.70
CPP	-3.42 ***	-5.31 – -1.53	-3.55
Trial Number	-0.35 ***	-0.55 – -0.15	-3.36
Random Effects			
σ^2	3.29		
$\tau_{00,P}$	1.19		
$\tau_{11,P.curTrial}$	0.43		
$\tau_{11,P.pCP}$	47.86		
ρ_{01}	-0.39		
	0.59		
ICC	0.57		
N_P	194		
Observations		2505	
Marginal R^2/Conditional R^2		0.084 / 0.607	
			*p<0.05 ** p<0.01 *** p<0.001

			ST3 Experiment 1 - SIWF
Predictors	Log-Odds	CI	Statistic
Intercept	0.42 ***	0.28 – 0.56	5.90
SIWF	-0.16 ***	-0.22 – -0.09	-4.46
Latency	-0.50 ***	-0.56 – -0.44	-15.87
Trial Number	-0.36 ***	-0.43 – -0.28	-9.80
SIWF x Latency	-0.04	-0.11 – 0.03	-1.15
Random Effects			
σ^2	3.29		
$\tau_{00,P}$	1.40		
$\tau_{11,P.Latency}$	0.13		
$\tau_{11,P.SIWF}$	0.07		
ρ_{01}	0.10		
	-0.25		
ICC	0.32		
N_P	352		
Observations		7040	
Marginal R^2/Conditional R^2		0.087 / 0.378	
			*p<0.05 ** p<0.01 *** p<0.001

			ST3 Experiment 2 - SIWF
Predictors	Log-Odds	CI	Statistic
Intercept	-0.44 **	-0.77 – -0.11	-2.63
SIWF	-0.41 **	-0.66 – -0.15	-3.14
Latency	0.57 ***	0.44 – 0.71	8.53
Trial Number	-0.03	-0.16 – 0.10	-0.44
SIWF x Latency	0.11	-0.07 – 0.29	1.24
Random Effects			
σ^2	3.29		
$\tau_{00,P}$	4.24		
$\tau_{11,P.Latency}$	0.03		
$\tau_{11,P.SIWF}$	0.05		
ρ_{01}	0.11		
	-0.29		
ICC	0.56		
N_P	191		
Observations		2424	
Marginal R^2/Conditional R^2		0.062/0.589	
			*p<0.05 ** p<0.01 *** p<0.001

Supplemental Table 4. Experiment 5 model outputs

			**ST3 Experiment 3 - SIWF**
Predictors	Log-Odds	CI	Statistic
Intercept	0.52 ***	0.27 – 0.76	4.15
SIWF	-0.50 ***	-0.64 – -0.35	-6.79
Latency	0.39 ***	0.27 – 0.50	6.50
Trial Number	0.06	-0.07 – 0.18	0.90
SIWF x Latency	-0.01	-0.13 – 0.11	-0.22
Random Effects			
σ^2	3.29		
$\tau_{00,P}$	2.27		
$\tau_{11,P.Latency}$	0.06		
$\tau_{11,P.SIWF}$	0.02		
ρ_{01}	-0.25		
	-0.87		
ICC	0.40		
N_P	191		
Observations		2433	
Marginal R^2/Conditional R^2		0.053 / 0.434	
			***p<0.05 ** p<0.01 *** p<0.001**

			**ST3 Experiment 4 - SIWF**
Predictors	Log-Odds	CI	Statistic
Intercept	2.22 ***	1.97 – 2.46	17.89
SIWF	-1.84 ***	-2.63 – -1.06	-4.59
Trial Number	-0.03	-0.24 – 0.18	-0.30
Random Effects			
σ^2	3.29		
$\tau_{00,P}$	1.26		
$\tau_{11,P.curTrial}$	0.24		
$\tau_{11,P.SIWF}$	7.33		
ρ_{01}	-0.28		
	0.20		
ICC	0.60		
N_P	194		
Observations		2505	
Marginal R^2/Conditional R^2		0.256 / 0.663	
			***p<0.05 ** p<0.01 *** p<0.001**

			**ST4 Experiment 5 – CPP no interaction**
Predictors	Log-Odds	CI	Statistic
Intercept	0.81 ***	0.59 – 1.02	7.39
CPP	-0.28 *	-0.54 – -0.02	-2.13
Latency	-0.19 ***	-0.21 – -0.18	-24.43
Run Number	0.00	-0.00 – 0.00	0.54
CPP x Latency	0.00	-0.07 – 0.08	0.13
Random Effects			
σ^2	3.29		
$\tau_{00,P}$	2.03		
ICC	0.38		
N_P	195		
Observations		26196	
Marginal R^2/Conditional R^2		0.028/0.399	
			***p<0.05 ** p<0.01 *** p<0.001**

ST4 Experiment 5 – CPP x Run			
Predictors	Log-Odds	CI	Statistic
Intercept	0.82 ***	0.61 – 1.04	7.51
CPP	-0.51 **	-0.86 – -0.15	-2.81
Latency	-0.19 ***	-0.21 – -0.18	-24.42
Run Number	-0.00	-0.00 – 0.00	-0.06
CPP x Latency	0.00	-0.07 – 0.07	0.04
CPP x Run	0.01	-0.00 – 0.03	1.83
Random Effects			
σ^2	3.29		
$\tau_{00,P}$	2.03		
ICC	0.38		
N_P	195		
Observations		26196	
Marginal R^2/Conditional R^2		0.028 / 0.399	
***p<0.05 ** p<0.01 *** p<0.001**			

ST4 Experiment 5 – SIWF no interaction			
Predictors	Log-Odds	CI	Statistic
Intercept	0.81 ***	0.59 – 1.02	7.37
SIWF	-0.33 *	-0.59 – -0.08	-2.56
Latency	-0.19 ***	-0.21 – -0.18	-25.16
Run Number	0.00	-0.00 – 0.00	0.53
SIWF x Latency	0.02	-0.05 – 0.09	0.46
Random Effects			
σ^2	3.29		
$\tau_{00,P}$	2.03		
ICC	0.38		
N_P	195		
Observations		26196	
Marginal R^2/Conditional R^2		0.028 / 0.399	
***p<0.05 ** p<0.01 *** p<0.001**			

ST4 Experiment 5 – SIWF x Run			
Predictors	Log-Odds	CI	Statistic
Intercept	0.82 ***	0.60 – 1.03	7.45
SIWF	-0.56 **	-0.91 – -0.21	-3.11
Latency	-0.19 ***	-0.21 – -0.18	-25.17
Run Number	0.00	-0.00 – 0.00	0.13
SIWF x Latency	0.01	-0.06 – 0.08	0.37
SIWF x Run	0.01	-0.00 – 0.03	1.82
Random Effects			
σ^2	3.29		
$\tau_{00,P}$	2.03		
ICC	0.38		
N_P	195		
Observations		26196	
Marginal R^2/Conditional R^2		0.028/0.399	
***p<0.05 ** p<0.01 *** p<0.001**			

Supplemental Table 5. Experiment 6 model outputs

	ST5 Experiment 6a - Null		
Predictors	Log-Odds	CI	Statistic
Intercept	0.52 ***	0.40 – 0.63	8.87
Trial Number	-0.36 ***	-0.42 – -0.30	-11.65
Experiment	-0.30 ***	-0.41 – -0.18	-5.07
Latency	-0.52 ***	-0.57 – -0.48	-23.45
Repetition	-0.15 ***	-0.22 – -0.07	-3.99
Random Effects			
σ^2	3.29		
$\tau_{00,P}$	1.32		
$\tau_{11,P.Latency}$	0.17		
$\rho_{01,P}$	0.14		
ICC	0.31		
N_P	552		
Observations		12541	
Marginal R^2/Conditional R^2		0.086 / 0.373	
			*p<0.05 ** p<0.01 *** p<0.001

	ST5 Experiment 6a - Surprise		
Predictors	Log-Odds	CI	Statistic
Intercept	0.55 ***	0.44 – 0.67	9.28
Trial Number	-0.33 ***	-0.39 – -0.27	-10.32
Experiment	-0.29 ***	-0.40 – -0.17	-4.89
Latency	-0.52 ***	-0.57 – -0.48	-23.40
Surprise	-0.18 **	-0.30 – -0.06	-3.04
Repetition	-0.13 ***	-0.20 – -0.06	-3.57
Random Effects			
σ^2	3.29		
$\tau_{00,P}$	1.32		
$\tau_{11,P.curTrial}$	0.17		
$\rho_{01,P}$	0.15		
ICC	0.31		
N_P	552		
Observations		12541	
Marginal R^2/Conditional R^2		0.087 / 0.374	
			*p<0.05 ** p<0.01 *** p<0.001

	ST5 Experiment 6a - Interaction		
Predictors	Log-Odds	CI	Statistic
Intercept	0.55 ***	0.44 – 0.67	9.27
Trial Number	-0.33 ***	-0.39 – -0.27	-10.30
Experiment	-0.29 ***	-0.40 – -0.17	-4.89
Latency	-0.51 ***	-0.56 – -0.46	-21.21
Surprise	-0.18 **	-0.29 – -0.06	-2.95
Repetition	-0.13 ***	-0.21 – -0.06	-3.58
Latency x Surprise	-0.08	-0.20 – 0.04	-1.23
Random Effects			
σ^2	3.29		
$\tau_{00,P}$	1.32		
$\tau_{11,P.curTrial}$	0.17		
$\rho_{01,P}$	0.15		
ICC	0.31		
N_P	552		
Observations		12541	
Marginal R^2/Conditional R^2		0.087/0.374	
			*p<0.05 ** p<0.01 *** p<0.001

ST5 Experiment 6b - Null			
Predictors	Log-Odds	CI	Statistic
Intercept	0.02	-0.21 – 0.24	0.13
Trial Number	-0.13 **	-0.21 – -0.05	-3.18
Experiment	-0.75 ***	-0.99 – -0.51	-6.16
Latency	0.30 ***	0.22 – 0.37	7.66
Repetition	-0.26 ***	-0.38 – -0.14	-4.26
Random Effects			
σ^2	3.29		
$\tau_{00,P}$	4.30		
$\tau_{11,P.Latency}$	0.06		
$\rho_{01,P}$	-0.22		
ICC	0.57		
N_P	389		
Observations		4985	
Marginal R^2/Conditional R^2		0.055 / 0.592	
			p<0.05 ** p<0.01 *** p<0.001

ST5 Experiment 6b - Surprise			
Predictors	Log-Odds	CI	Statistic
Intercept	0.07	-0.16 – 0.30	0.61
Trial Number	-0.04	-0.13 – 0.06	-0.72
Experiment	-0.81 ***	-1.06 – -0.56	-6.42
Latency	0.30 ***	0.22 – 0.38	7.48
Surprise	-0.49 ***	-0.76 – -0.22	-3.57
Repetition	-0.32 ***	-0.46 – -0.19	-4.82
Random Effects			
σ^2	3.29		
$\tau_{00,P}$	4.32		
$\tau_{11,P.curTrial}$	0.06		
$\rho_{01,P}$	-0.14		
ICC	0.57		
N_P	389		
Observations		4985	
Marginal R^2/Conditional R^2		0.058 / 0.595	
			p<0.05 ** p<0.01 *** p<0.001

ST5 Experiment 6b - Interaction			
Predictors	Log-Odds	CI	Statistic
Intercept	0.07	-0.15 – 0.30	0.62
Trial Number	-0.04	-0.13 – 0.06	-0.73
Experiment	-0.82 ***	-1.06 – -0.57	-6.54
Latency	0.29 ***	0.21 – 0.37	6.92
Surprise	-0.49 ***	-0.75 – -0.23	-3.71
Repetition	-0.33 ***	-0.46 – -0.20	-5.01
Latency x Surprise	0.08	-0.14 – 0.31	0.75
Random Effects			
σ^2	3.29		
$\tau_{00,P}$	4.31		
$\tau_{11,P.curTrial}$	0.06		
$\rho_{01,P}$	-0.12		
ICC	0.57		
N_P	389		
Observations		4985	
Marginal R^2/Conditional R^2		0.058/0.595	
			p<0.05 ** p<0.01 *** p<0.001
